# Metastatic meningioma presenting as a malignant soft tissue tumour

**DOI:** 10.1186/s13569-016-0063-1

**Published:** 2016-12-30

**Authors:** Catherine McCarthy, Monika Hofer, Marianna Vlychou, Robar Khundkar, Paul Critchley, Simon Cudlip, Olaf Ansorge, Nick A. Athanasou

**Affiliations:** 1 Department of Radiology, Nuffield Orthopaedic Centre, Windmill Road, Oxford, OX3 7HE UK; 2 Department of Neuropathology, Oxford University Hospitals Oxford, Oxford, OX3 9DU UK; 3 Nuffield Department of Orthopaedics, Rheumatology and Musculoskeletal Sciences, Nuffield Orthopaedic Centre, University of Oxford, Windmill Road, Oxford, OX3 7HE UK; 4 Sarcoma Service, Nuffield Orthopedic Centre, Windmill Road, Oxford, OX3 7HE UK; 5 Department of Neurosurgery, Oxford University Hospitals, Oxford, OX3 9DU UK

**Keywords:** Meningioma anaplastic, Metastasis, Soft tissue, Tumour

## Abstract

**Background:**

Extracranial metastasis of malignant meningioma to soft tissues is extremely rare and its clinical, radiological and pathological features are not well-characterised.

**Case presentation:**

We report a case of a 58 year old man who presented with a mobile mass within the left trapezius muscle. The patient had previously undergone surgery for a right frontal lobe high grade anaplastic meningioma. Histology of the soft tissue lesion showed metastatic anaplastic meningioma with clumps of pleomorphic tumour cells which expressed epithelial membrane antigen, cytokeratin and P63 but were negative for other epithelial and mesenchymal markers. A PET-CT scan revealed additional metastatic lesions in the left pleura, liver and iliac bone.

**Conclusions:**

Metastatic malignant meningioma can very rarely present as a high grade pleomorphic malignant soft tissue tumour and needs to be distinguished from soft tissue sarcomas and metastatic carcinomas that express epithelial antigens.

## Background

Meningiomas are relatively common primary intracranial tumours that arise within the leptomeninges or dura mater; these tumours exhibit morphological and immunophenotypic evidence of origin from meningothelial cells which are present in the arachnoid membrane and arachnoid villi associated with the intradural venous sinuses and their tributaries [1]. The majority of meningiomas arise within the cranial cavity and are supratentorial dura-based lesions that develop most commonly in the vicinity of the superior sagittal sinus, over the cerebral convexities or in contact with the falx cerebri. Approximately 12% arise in the spine.

Meningiomas show considerable morphologic variation and are most commonly benign tumours (Grade I) [2, 3]. Atypical (Grade II) and anaplastic (Grade III) meningiomas, which have aggressive/malignant potential, are distinguished on the basis of mitotic activity and a number of other features (e.g. pleomorphism, cellularity, necrosis). Anaplastic meningiomas commonly recur and very rarely have been known to metastasise to extracranial tissues and organs [3–5]. The true prevalence of metastatic malignant meningioma is unknown as many reported cases and case series include cases of “benign metastasising meningioma”, as well as tumours that were in the past incorrectly classified as meningiomas (e.g. angioblastic meningioma which is now known to be a solitary fibrous tumour).

The most frequently reported sites of extracranial metastasis of malignant meningioma are lung, spine and liver [3–13]. There are very few reports of malignant meningioma metastasising to deep soft tissues [4, 14–16]. We report a case of a metastatic anaplastic meningioma which presented as a growing soft tissue tumour in the trapezius muscle and review the literature regarding soft tissue involvement by metastatic meningioma.

## Case presentation

**Fig. 1:**
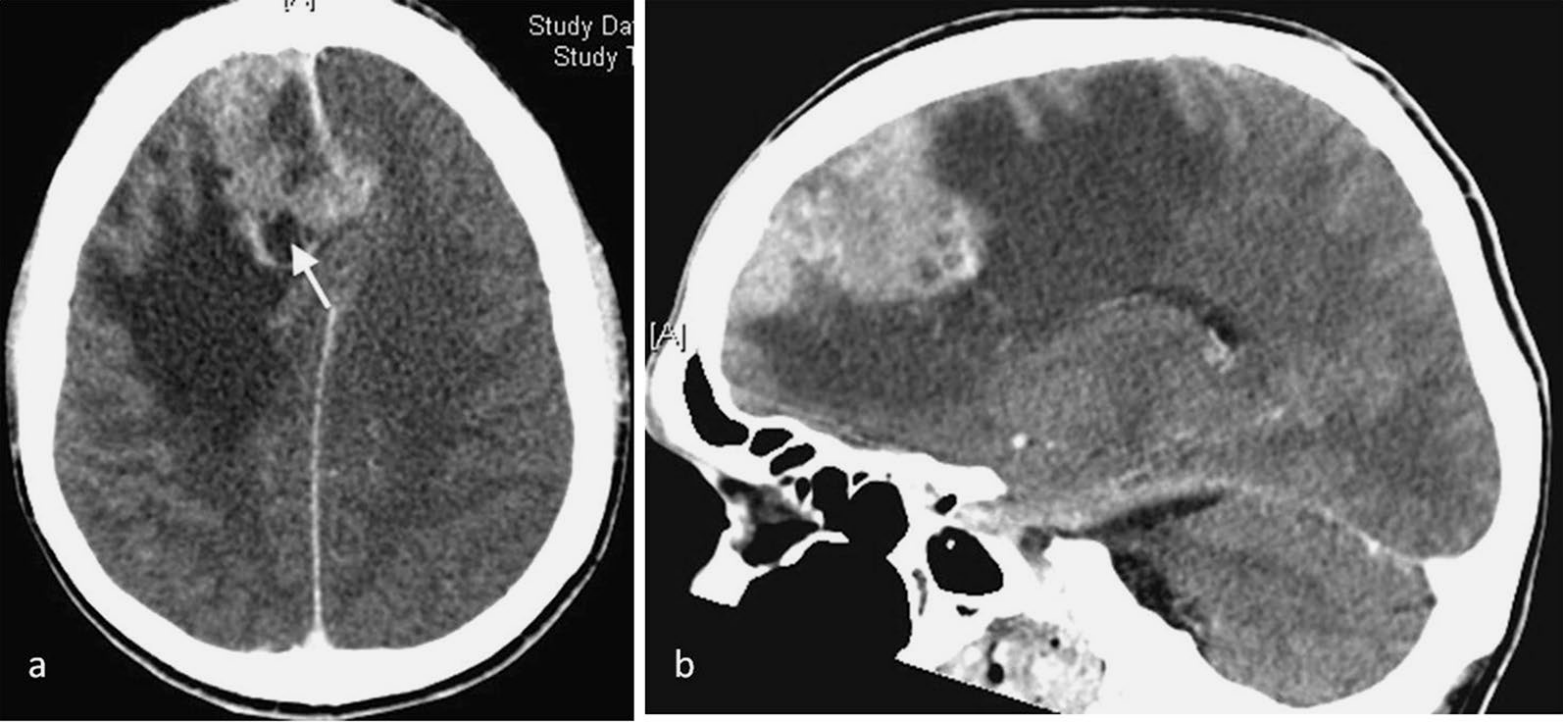
Axial CT post contrast image (**a**) and sagittal reconstruction (**b**) shows an irregular extra-axial solid mass with heterogeneous enhancement and deep cystic change (*arrow*) adjacent to the falx extending into the right frontal lobe. There is significant peritumoral oedema and mass effect involving the right frontal lobe with displacement of the midline. These findings, together with tumour interdigitating with the brain substance, are consistent with an aggressive frontal meningioma

A 55 year old man was admitted to hospital with a head injury that resulted in hearing loss on the left side with pulsatile tinnitus and a vague feeling of disorientation. A CT scan showed an irregular extra-axial solitary lesion lying adjacent to the falx on the right of the midline measuring 3 × 4 × 6 cm with significant mass-effect and peritumoral oedema involving most of the right frontal lobe. There was evidence of underlying bone remodelling with vascular dural supply and heterogeneous enhancement after contrast administration with a deep cystic component. There were fringes of tumour interdigitating with the brain substance indicating probable brain invasion (Fig. 1). The findings were consistent with an aggressive frontal meningioma. Partial surgical removal of the lesion in the right frontal lobe was undertaken. On histological examination, the lesion was classified as an atypical (WHO Grade II) meningotheliomatous meningioma.

**Fig. 2:**
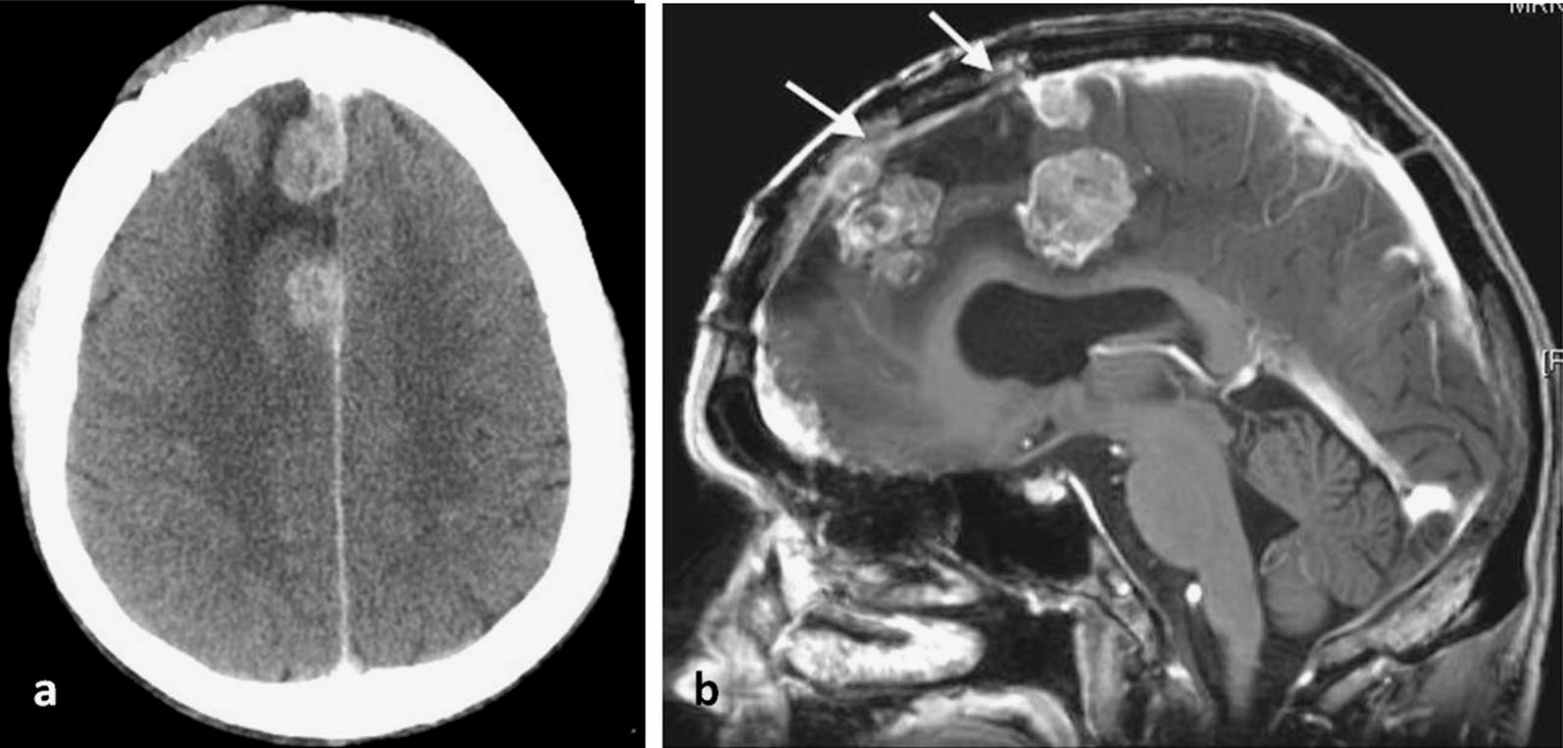
Axial CT post contrast image showing: **a** nodular enhancing masses and perilesional oedema in the right frontal lobe, indicative of local recurrence of the meningioma. **b** A sagittal T1-weighted MR image post contrast confirming the presence of three lesions and invasion of the superior sagittal sinus (*arrows*)

**Fig. 3:**
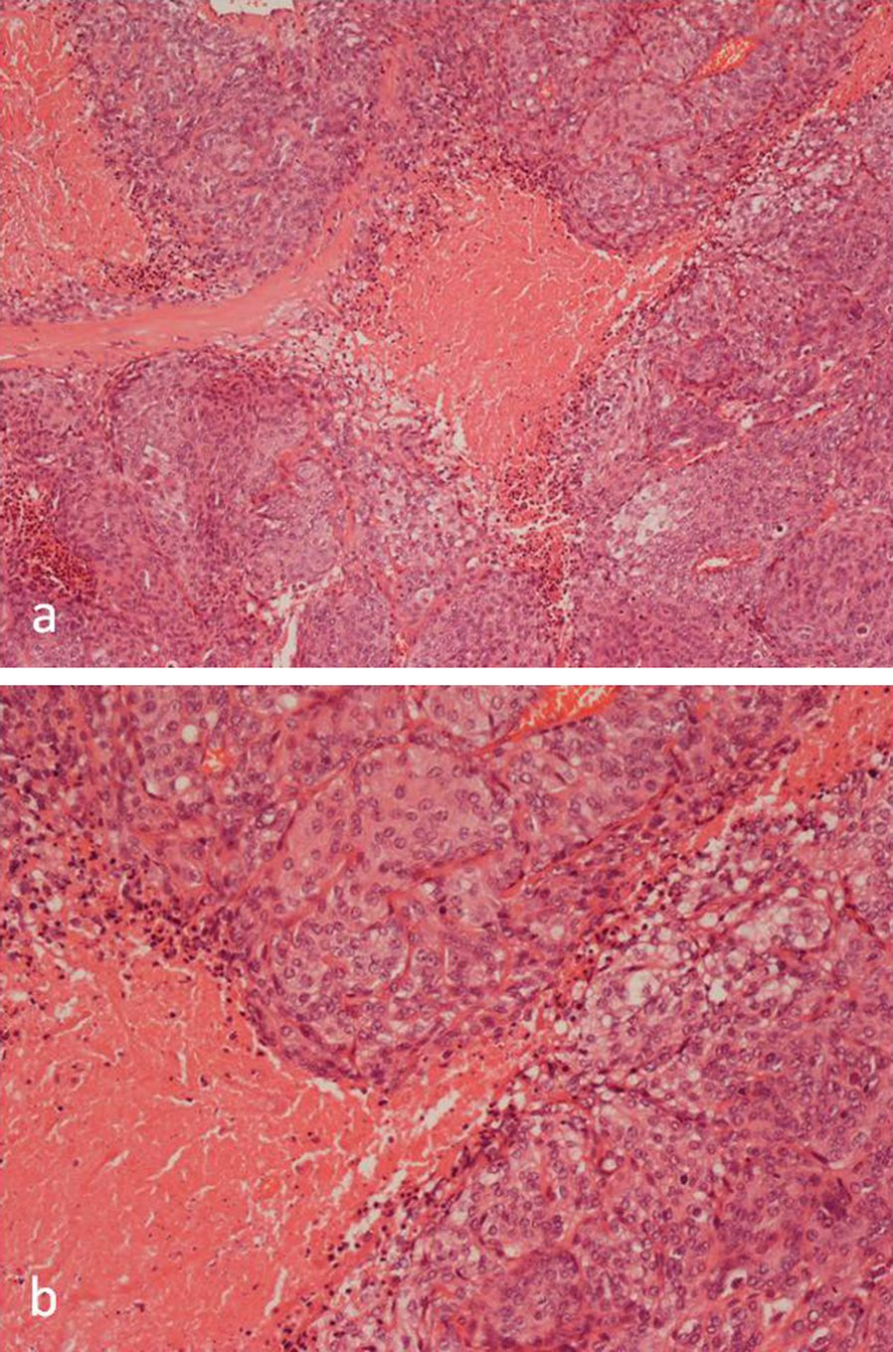
Histology of the anaplastic (Grade III) meningioma of the frontal lobe showing; **a** lobules of meningotheliomatous tumour containing cells with round vesicular nuclei, **b** areas of focal tumour necrosis containing more pleomorphic tumour cells

A year later, the patient experienced an episode of seizures and a CT scan revealed three new lesions at the site of the previously excised meningioma with extensive perilesional oedema. An MRI confirmed the CT findings indicative of tumour and also detected invasion and occlusion of the anterior part of the superior sagittal sinus (Fig. 2). Partial resection of the recurrent mass was again undertaken. Histology of the tumour specimen showed features of an anaplastic (WHO Grade III) meningotheliomatous meningioma with lobules of tumour containing cells with round or oval vesicular nuclei. Focally, there were cells which showed more prominent nuclear pleomorphism. There was increased mitotic activity and extensive tumour necrosis was noted (Fig. 3). Post-operative follow-up revealed residual tumour in the anterior part of the superior sagittal sinus, along the falx and the right frontal convexity. Follow-up imaging also confirmed interval progression of the residual meningioma.

**Fig. 4:**
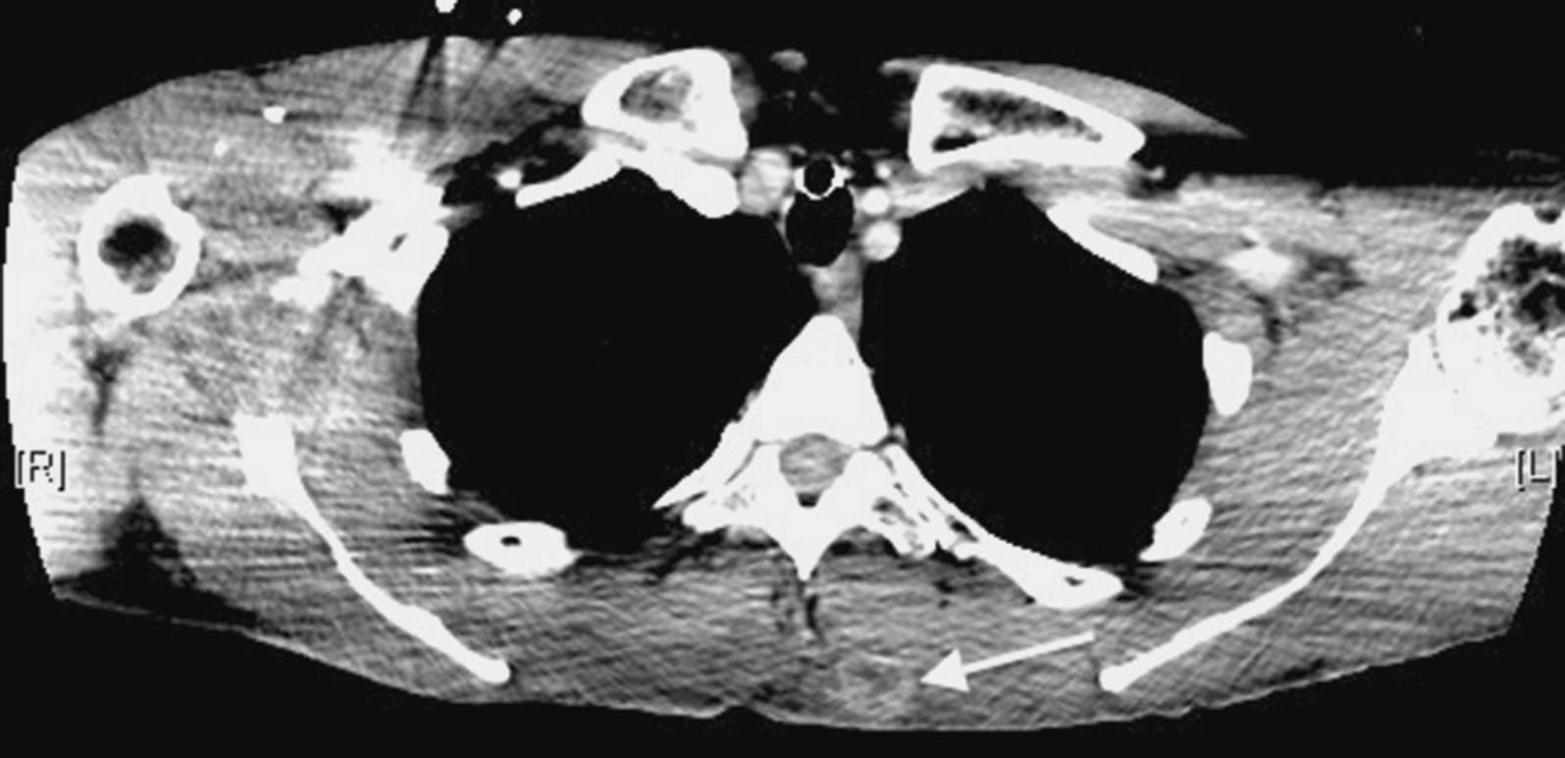
Axial thorax CT post contrast image showing an ill-defined soft tissue mass (*arrow*) superficially located in the left trapezius muscle with peripheral enhancement and low signal in the centre

**Fig. 5:**
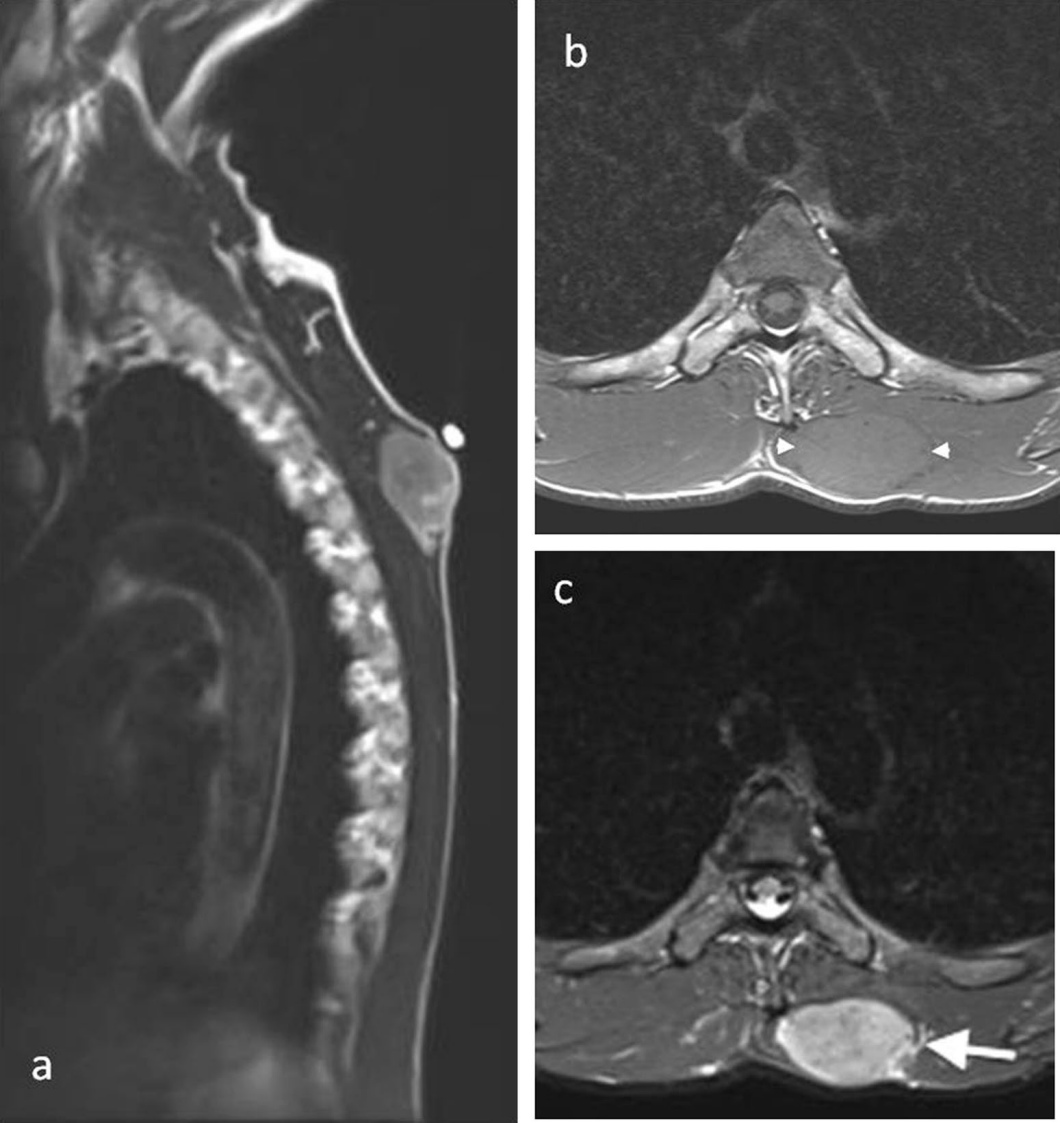
Sagittal T2 (**a**), axial T1 (**b**) and axial T2 fat saturated (**c**) MR images demonstrate a solid well defined soft tissue mass in the medial left trapezius muscle which returns isointense T1-W (between *arrow heads*) and heterogeneous predominantly high T2-W signal relative to skeletal muscle. There is mass effect on the deeper paravertebral muscles and peripheral feeding vessels along the lateral aspect of the lesion (*arrow*)

**Fig. 6:**
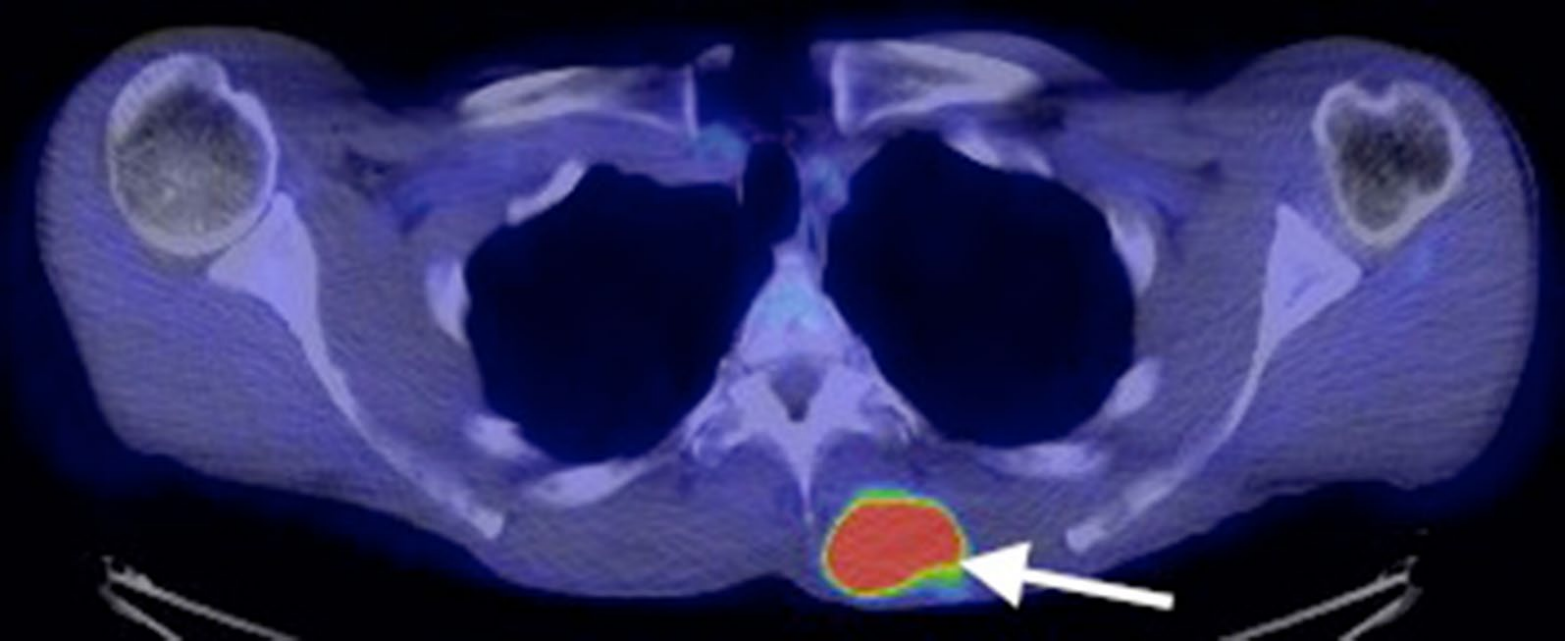
PET-CT confirms the presence of an FDG avid soft tissue mass in the left trapezius muscle

The patient completed a course of radiotherapy and, nine months later, a mobile soft tissue lump on his back was noted. A thoracic CT scan revealed a soft tissue lesion superficially located in the inferior portion of the left trapezius muscle. The lesion was lobulated, had a low attenuation centre, showed peripheral enhancement and did not exhibit evidence of matrix calcification (Fig. 4). The lesion increased in size and a subsequent MRI scan performed 6 months later confirmed the presence of a solid soft tissue mass up to 5 cm in diameter in the medial left trapezius muscle. The lesion indented the underlying paravertebral muscles but remained well-defined and did not invade the deeper musculature. The lesion returned isointense T1-W signal relative to skeletal muscle and heterogeneous high T2-W signal. There was no internal calcification, haemorrhage or cystic degeneration. Peripheral feeding vessels were present (Fig. 5). A PET-CT scan demonstrated that the intramuscular lesion was extremely FDG avid with an SUVmax of 22.1 (Fig. 6). PET-CT also revealed disseminated, markedly FDG avid metastases in liver, bone and pleura and residual meningioma in the right frontal lobe.

**Fig. 7:**
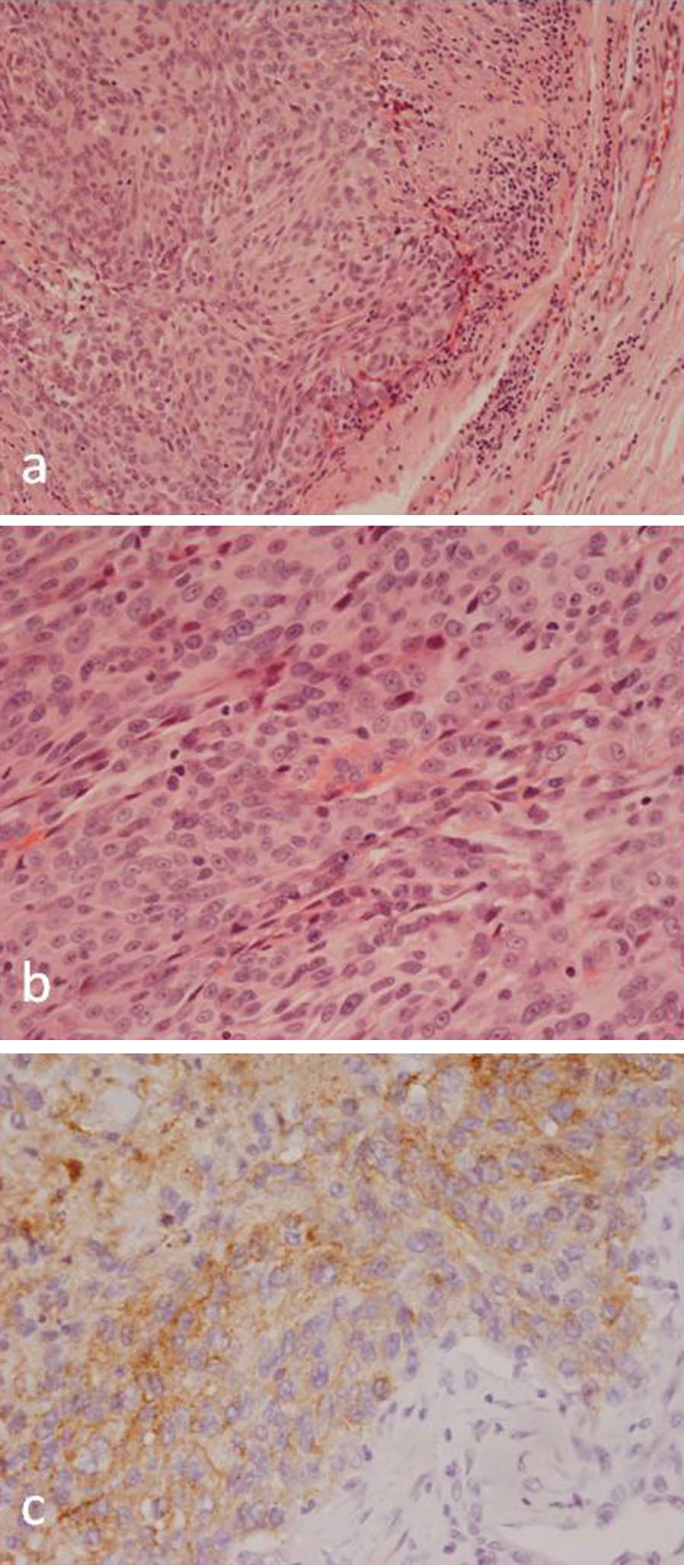
Histology of the metastatic meningioma in soft tissues showing: **a** lobules of tumour showing features of meningotheliomatous meningioma similar to the primary brain tumour **b** areas where tumour cells exhibit marked nuclear pleomorphism and contain prominent nucleoli. **c** Expression of EMA by tumour cells

The lesion was biopsied and showed a largely lobulated tumour containing collections of tumour cells, many of which showed similar features to those seen in the frontal lobe tumour. There were a few whorled collections of tumour cells with round or oval vesicular nuclei (Fig. 7). There was prominent nuclear pleomorphism, a high mitotic rate and focal tumour necrosis. Focally there were solid areas of proliferation of pleomorphic tumour cells, some of which had vacuolated cytoplasm. Immunohistochemistry showed strong expression of epithelial membrane antigen (EMA) and P63; there was also focal expression of cytokeratin (CK7+, CK20−), nuclear staining for IN-1, and a high Ki-67 fraction. There was no expression of desmin, S100, CD10, CD30, CD68, myogenin, smooth muscle actin, estrogen receptor, progesterone receptor, chromogranin, carcinoembryonic antigen or TTF1. There was initially some difficulty in establishing the pathological diagnosis as the previous history of recurrent atypical/anaplastic meningioma was not provided to the reporting pathologist whose initial differential diagnosis included a soft tissue metastasis of carcinoma and a primary soft tissue sarcoma expressing the epithelial markers. However, once the history of meningioma and the radiological findings were taken into account, it was clear that the soft tissue mass represented a metastasis of the previously diagnosed anaplastic meningotheliomatous meningioma.

A wide local excision of the lesion, which measured 5 × 3 × 2 cm, was performed; this included a cuff of normal muscle around the lesion and a 1 cm margin of skin around the previous biopsy scar. Histological findings in the resection specimen were similar to those of the biopsy. Further follow up of the patient by CT scan showed no recurrence of the soft tissue lesion but progression of disease in the brain.

## Discussion

Extracranial metastasis of meningioma occurs rarely with only a few case reports documenting deep soft tissue as a site of metastasis [4, 14–17]; it is not clear from many of these reports whether, as in our case, the metastasis involved soft tissue alone or whether other contiguous structures, such as lymph nodes and bone, were also involved. Doxtader et al. [14] described an aggressive meningioma in an 8 year old patient with metastasis to cervical soft tissue and lymph nodes. There is also a report of an isolated “chest wall” metastasis and of a bone/soft tissue metastasis from an atypical primary meningioma of the nasal septum that clinically simulated a soft tissue sarcoma [15, 16]. In a review of the literature between 1886 and 1958, Karasick et al. [5] reported 56 patients who developed extracranial meningioma metastasis, none of which were in soft tissue. In the database review of Enam et al. [9], the incidence of meningioma metastasis was 0.76% with no soft tissue metastasis being noted. Forest et al. [3] also carried out a database review totalling 1291 meningiomas and identified only four cases of metastatic meningioma, none of which was in soft tissues.

A recent review of the literature on all reports of distant metastasis of intracranial meningiomas found that in 115 patients with 164 metastatic lesions, 83.9% were Grade I (benign), 20.0% were Grade II (atypical) and 40% Grade III (anaplastic) meningiomas [4]. This analysis excluded pathological entities such as “angioblastic meningioma”, meningeal carcinomas and sarcomas, all of which were formerly considered to represent subtypes of meningioma. In this review, 6% of cases developed in cervical soft tissue, 2.4% in skin and subcutaneous tissue and 1.8% in muscle. Haematogenous spread of meningiomas through the paravertebral and jugular venous systems, which also drain thoracic tissues, has been postulated and could account for the relatively high frequency of the neck and upper trunk as a site of soft tissue and lymph node metastasis. Although anaplastic and atypical meningiomas were most commonly associated with metastatic lesions, comprising 31.3 and 19.1% of cases respectively, it was noted that Grade I meningiomas can also give rise to metastatic lesions. It was also noted that in 93% of cases the intracranial meningioma was diagnosed and resected before the distant metastasis appeared. The prognosis of intracranial meningioma is determined by the histological grade [1, 2], and in our case progression of the intracranial tumour to a higher histological grade was noted.

Radiological features of the chest wall tumour in our case were not specific; the diagnosis of a metastatic tumour was supported radiologically by the interval increase in the size of the lesion, as well as high FDG uptake and the finding of lesions at other sites on the PET-CT scan. Multiple deposits were also observed in other extracranial sites in previous reports of soft tissue metastasis from malignant meningioma [3, 4]. The tumour in the frontal lobe showed several radiological findings that have been described as potential markers of atypical or malignant meningiomas including indistinct or irregular tumour margins, marked peritumoral oedema, inhomogeneous enhancement post-contrast, intrinsic cyst-like areas, disruption of the arachnoid at the brain-tumour interface and adjacent bone destruction [18–24].

Histological features of the chest wall tumour showed that it was focally necrotic and contained collections of tumour cells with elongated cytoplasm and round or oval vesicular nuclei; there was prominent nuclear pleomorphism and increased mitotic activity. Morphologically, the differential diagnosis was wide and included sarcoma, lymphoma, metastatic melanoma and carcinoma. Immunohistochemistry showed expression of epithelial markers on tumour cells with diffuse, strong staining for EMA and focal staining for cytokeratin. In the absence of any previous history, and taking into account other immunohistochemical findings, these features were thought to favour metastatic carcinoma or a sarcoma expressing epithelial markers. Several sarcomas are known to express epithelial markers, including synovial sarcoma, epithelioid sarcoma, epithelioid malignant peripheral nerve sheath tumour, myoepithelioma and leiomyosarcoma [25] Morphological and immunophenotypic features were not typical of any of these specific tumour types. Soft tissue metastasis of carcinoma is well recognised with autopsy series reporting that this occurs in 0.75–9% of patients who die of metastatic carcinoma [26–29]. Lung is the most common primary carcinoma that results in soft tissue metastasis with a mean prevalence of 2.3% being reported [30], and the cytokeratin profile in our case was consistent with a metastatic lung carcinoma; it was not typical for metastatic renal or colon carcinoma, both which have also been reported to produce soft tissue metastasis with relative frequency. Although metastatic malignant meningioma is much less common than metastatic carcinoma, the prevalence of metastatic meningioma in skin, subcutaneous tissue and muscle is, at least in one case series [4], comparable to that of metastatic carcinoma. In our case the diagnosis of metastatic meningioma became clear when the full past history of the patient was recognised. It could be argued that the possibility of malignant meningioma should have been suspected earlier given that the morphological and immunohistochemical findings were so typical of this tumour with focal, occasionally whorled collections of strongly EMA+ tumour cells being typical of a meningotheliomatous meningioma [1, 2]. It should be noted that in more diagnostically challenging cases certain genetic/cytogenetic abnormalities such as inactivating mutations of NF2, monosomy 22, as well as specific alterations in gene expression, may also be useful in establishing the diagnosis of metastatic meningioma [31].

## Conclusion

Knowledge of the clinical and radiological information was essential to establish the diagnosis of metastatic malignant meningioma in our case, particularly the previous history of anaplastic meningioma and the radiological identification of FDG avid lesions at several sites. Although very rare, the possibility of metastatic meningioma should be considered in the differential diagnosis of a malignant soft tissue tumour, where tumour cells strongly express EMA and other epithelial markers.

## Abbreviations

PET: positron-emission tomography
FDG: fludeoxyglucose
EMA: epithelial membrane antigen
WHO: World Health Organization
CT: computed tomography
MRI: magnetic resonance imaging
CK: cytokeratin
